# Quantifying optical anisotropy in soft tissue membranes using Mueller matrix imaging

**DOI:** 10.1117/1.JBO.26.10.106001

**Published:** 2021-10-06

**Authors:** Alexander W. Dixon, Andrew J. Taberner, Martyn P. Nash, Poul M. F. Nielsen

**Affiliations:** a University of Auckland, Auckland Bioengineering Institute, Auckland, New Zealand; b University of Auckland, Department of Engineering Science, Auckland, New Zealand

**Keywords:** optical polarization, Mueller matrix imaging, tissue anisotropy, collagen, bioprosthetic heart valves

## Abstract

**Significance:** A non-destructive technique for accurately characterizing the spatial distribution of optical properties of soft tissue membranes may give improved outcomes in many tissue engineering applications.

**Aim:** This study aimed to develop a non-destructive macroscopic imaging technique that is sensitive to optical anisotropy, typical of fibrous components in soft tissue membranes, and can address some of the difficulties caused by the complex turbid nature of these tissues.

**Approach:** A near-infrared Mueller matrix imaging polarimeter employing logarithm decomposition was developed and used to conduct transmission measurements of all the polarization properties across the full thickness of bovine pericardium tissue.

**Results:** The full Mueller matrix was measured across a 70  mm×70  mm sample of calf bovine pericardium and revealed significant retardance (linear and circular) and depolarization in this tissue. Regions with a uniform axis of optical anisotropy were identified. Mueller matrix imaging demonstrated that the exhibited circular retardance was sufficient to lead to possible misinterpretation of apparent fiber orientation when using conventional polarization imaging techniques for such tissues.

**Conclusions:** Mueller matrix imaging can identify regional distributions of optical anisotropy in calf bovine pericardium. This new capability is a promising development in non-destructive imaging for tissue selection.

## 1 Introduction

Soft tissue membranes are used in many important tissue engineering applications, such as in the construction of bioprosthetic heart valves for replacing diseased leaflets. The requirements of functional performance and durability challenges the use of these tissues in such applications. The collagen fiber architecture of soft tissue membranes dominates their mechanical function,^1^^,^^2^ and thus quantification of this architecture, on a macroscopic spatial scale, is important and is ideally performed in a non-destructive manner so as not to alter the mechanical properties. For many applications, this non-destructive requirement precludes the use of many established techniques that require histological sectioning, staining, or optical clearing for assessing tissue microstructure.^3^^,^^4^

A suitable non-destructive imaging modality may be realized with polarization-sensitive techniques that can measure the optical anisotropy arising in biological samples due to microstructural anisotropy of fibrous structures, such as collagen.^5^ Depth-resolved optical polarization techniques, such as polarization-sensitive optical coherence tomography, can provide high spatial resolution measurements of optical anisotropy in soft tissues.^6^^,^^7^ However, these methods are complex and expensive to scale up to the large lateral fields of view required for imaging at the macroscopic scale. Bulk tissue polarization imaging methods are, in general, more straightforward to implement, less expensive than optical sectioning methods, and can be readily scaled to large lateral fields of view.^8^

Mueller matrix imaging is a polarization imaging technique that determines a transfer function, which represents all of the fundamental polarization properties of a sample: namely, retardance, diattenuation, and depolarization.^9^ The full Mueller matrix can be measured to characterize, without prior information, the polarization properties of a complex turbid medium.^8^ Mueller matrix decomposition can then be performed to separate the effects of multiple polarization properties, if present. Mueller matrix imaging is suitable for initial characterization of a sample with unknown polarization properties prior to the use of polarization imaging techniques more specifically designed to measure a particular subset of fundamental polarization properties. This imaging technique has evolved from point-based optical polarimetry techniques and, due to recent developments in instrumentation and data analysis, has seen widespread application in the general field of tissue polarimetry.^7^^–^^11^ Recent studies include Mueller matrix microscopy imaging to measure polarization properties, decomposed with the differential Mueller matrix formalism, for analyzing tissue microstructure in human skin equivalents,^12^ and optical anisotropy in histological sections of myocardium^13^ and brain tissue.^14^

Soft tissue membranes of porcine and bovine pericardium are used in the construction of bioprosthetic heart valves.^15^^,^^16^ These membranes may be thin enough to allow the use of forward scattering (transmission) Mueller matrix imaging polarimetry to measure the accumulated polarization properties across the full thickness of the membrane. Type I collagen, the primary fibrous component in pericardium,^17^ exhibits a positive uniaxial intrinsic linear birefringence with an optic axis that aligns with the collagen fiber orientation.^5^^,^^18^ Measurements of optical anisotropy, given by the optic axis orientation and the magnitude of the linear retardance, have been demonstrated to correspond to the average fiber orientation^19^^,^^20^ and relative fiber anisotropy,^21^^,^^22^ respectively, in collagenous soft tissues. Optical anisotropy thus provides a measure of microstructural anisotropy in these membranes. In pericardium, the fibers are predominantly aligned parallel to the membrane surface^23^ and thus measurement of the in-plane fiber orientation is generally sufficient to estimate the orientation of the collagen fibers for such tissues.

In this study, we adopt the established technique of Mueller matrix imaging and introduce an imaging polarimeter suitable for measuring the Mueller matrices of soft tissue membranes with a novel application for tissue engineering. We have developed an imaging polarimeter capable of measuring the bulk Mueller matrix across soft tissue membranes at a macroscopic scale through the use of near-infrared wavelength light in a transmission arrangement. This polarimeter has been integrated with mechanical testing instrumentation to enable highly controlled optomechanical testing to elicit strain-induced changes in the birefringence of different sample types. We adopt the logarithm decomposition, using the differential Mueller matrix formalism for processing Mueller matrix images. In the present study, we investigate the suitability of this approach for measuring optical anisotropy in soft tissue membranes and identify and discuss some practical limitations. The experimental results showed these tissues exhibit strong circular retardance, and we thereby demonstrate that Mueller matrix imaging is necessary for estimating the optical anisotropy of these tissues.

## 2 Materials and Methods

### 2.1 Mueller Matrix Imaging Polarimeter

**Fig. 1:**
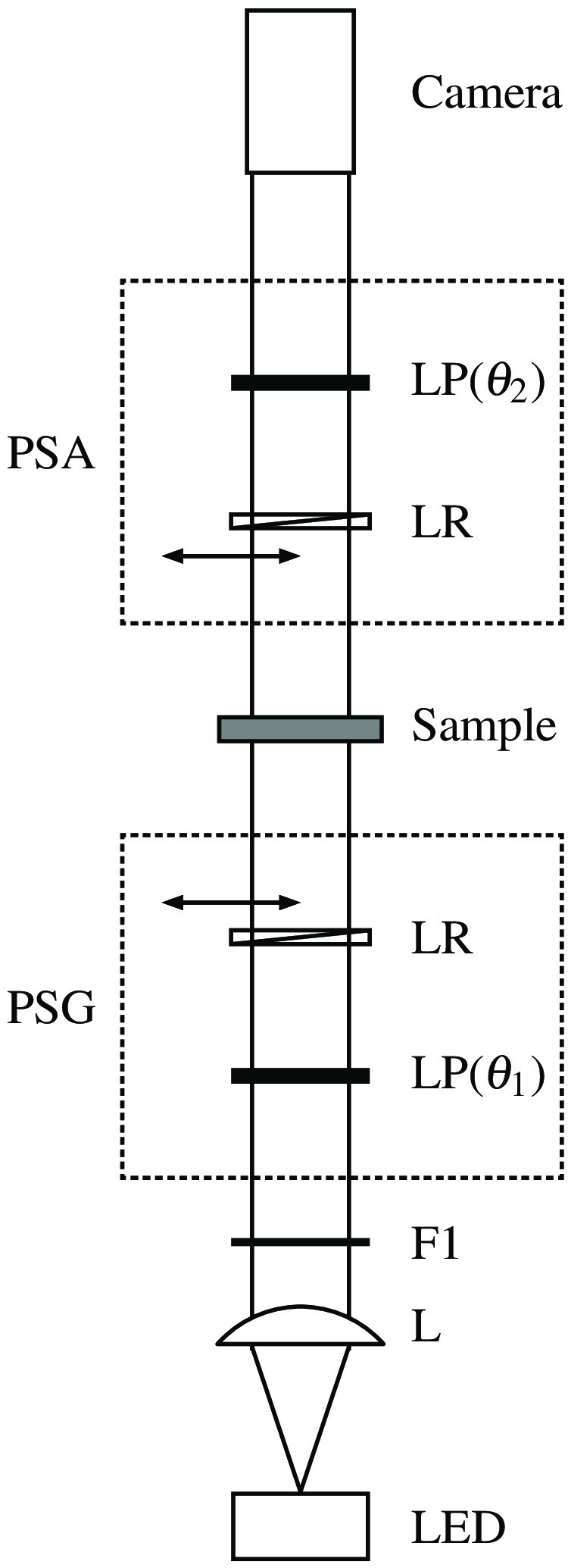
Schematic of the Mueller matrix imaging polarimeter. L: lens, F: filter, LP: linear polarizer, LR: linear retarder (quarter waveplate), PSG: polarization state generator, PSA: polarization state analyzer.

A schematic of the Mueller matrix imaging polarimeter is shown in Fig. 1. Light from a near-infrared LED with a center wavelength of 850 nm (Osram, SFH4715AS) was collimated and passed through a narrow bandpass filter with full-width at half maximum of 10 nm (Thorlabs, FL850-10). The light beam (20-mm diameter) next passed through a polarization state generator (PSG), through the sample, and then through a polarization state analyzer (PSA). The PSG and PSA each consisted of a linear polarizer (LP) (Thorlabs, LPVIS100) mounted in a motorized precision rotation stage (Thorlabs, PRM1/MZ8 and K10CR1) and an achromatic quarter waveplate (Thorlabs, AQWP10M) mounted in a motorized filter wheel (Thorlabs, FW102). The analyzer components were arranged in opposite order from the generator components. Light transmitted through the analyzer was focused onto a monochromatic camera (FLIR, BFS-U3-51S5M-C). Samples were inserted in the optical path between the PSG and the PSA and imaged with a field of view of 14 mm at a pixel resolution of 8.90  μm.

The configuration of the PSG and the PSA enabled generation and analysis of linear horizontal (H), linear vertical (V), linear +45  deg (P), and right circular (R) polarization states. Mueller matrix images (M) were calculated from 16 images according to

$$M=[\begin{array}{cccc} m_{11} & m_{12} & m_{13} & m_{14}\\ m_{21} & m_{22} & m_{23} & m_{24}\\ m_{31} & m_{32} & m_{33} & m_{34}\\ m_{41} & m_{42} & m_{43} & m_{44} \end{array}]=[\begin{array}{cccc} I_{\mathrm{HH}}+I_{\mathrm{HV}}+I_{\mathrm{VH}}+I_{\mathrm{VV}} & I_{\mathrm{HH}}+I_{\mathrm{HV}}-I_{\mathrm{VH}}-I_{\mathrm{VV}} & 2I_{\mathrm{PH}}+2I_{\mathrm{PV}}-m_{11} & 2I_{\mathrm{RH}}+2I_{\mathrm{RV}}-m_{11}\\ I_{\mathrm{HH}}-I_{\mathrm{HV}}+I_{\mathrm{VH}}-I_{\mathrm{VV}} & I_{\mathrm{HH}}-I_{\mathrm{HV}}-I_{\mathrm{VH}}+I_{\mathrm{VV}} & 2I_{\mathrm{PH}}-2I_{\mathrm{PV}}-m_{21} & 2I_{\mathrm{RH}}-2I_{\mathrm{RV}}-m_{21}\\ 2I_{\mathrm{HP}}+2I_{\mathrm{VP}}-m_{11} & 2I_{\mathrm{HP}}-2I_{\mathrm{VP}}-m_{12} & 4I_{\mathrm{PP}}-2I_{\mathrm{PH}}-2I_{\mathrm{PV}}-m_{31} & 4I_{\mathrm{RP}}-2I_{\mathrm{RH}}-2I_{\mathrm{RV}}-m_{31}\\ 2I_{\mathrm{HR}}+2I_{\mathrm{VR}}-m_{11} & 2I_{\mathrm{HR}}-2I_{\mathrm{VR}}-m_{12} & 4I_{\mathrm{PR}}-2I_{\mathrm{PH}}-2I_{\mathrm{PV}}-m_{41} & 4I_{\mathrm{RR}}-2I_{\mathrm{RH}}-2I_{\mathrm{RV}}-m_{41} \end{array}]$$ (1)

where I represents an image of the sample with the polarization state of the PSG and the PSA indicated by the first and second subscripts, respectively.^24^ Mueller matrix images were normalized to the transmission of unpolarized light ($m_{11}$ element) at each pixel. The polarimeter was controlled via custom LabVIEW software (National Instruments, 2018), enabling each of the images in Eq. (1) to be acquired in a fully automated manner. The acquisition time for the 16 measurements was ∼90  s.

### 2.2 Mueller Matrix Decomposition

For media that exhibit multiple polarization effects, the fundamental polarization properties are not always represented explicitly in the Mueller matrix. To separate the polarization properties, the Mueller matrix can be decomposed using one of a number of methods.^25^ For complex turbid media, such as soft tissues, multiple polarization properties are most likely exhibited simultaneously. The logarithm decomposition,^26^ using the differential Mueller matrix formalism,^27^ represents a medium as one that exhibits simultaneous polarization properties, as opposed to ordered serial application of polarization properties.

We adopted the logarithm decomposition and assumed the medium to be homogeneous across the imaging plane (within each pixel) and across the sample thickness. Two distinct physical cases of the Mueller matrix M were decomposed, where M had a complex conjugate pair of eigenvalues or where all eigenvalues of M were real and positive, which physically correspond to a Mueller matrix for a medium with or without birefringence (linear and/or circular), respectively.^28^ The former case was decomposed using the real Jordan normal form of M, as described by Devlaminck and Ossikovski.^29^ In this study, if any of the eigenvalues of M were real and negative, zero, or did not match the cases outlined above, then the subsequent calculated polarization properties were considered undefined. It is important to note that, if there is an unknown order (number of wavelengths) of total retardance occurring in the medium then the order of retardance must be determined, or carefully assumed, to obtain physically meaningful properties from the decomposition. The logarithm decomposition was implemented in MATLAB (MathWorks, R2020b) and performed on measured Mueller matrix images. The following accumulated polarization properties were calculated according to Kumar et al:^30^^,^^31^ the linear retardance $\delta_{L}$ $\left(0\leq \delta_{L}<\pi \right)$ and its fast axis orientation $\theta_{\mathrm{FA}}$; the circular retardance $\delta_{C}$ $\left(-\pi \leq \delta_{C}<\pi \right)$; the linear diattenuation $D_{L}$ $\left(0\leq D_{L}\leq 1\right)$ and its transmission axis orientation $\theta_{\mathrm{TA}}$; the circular diattenuation $D_{C}$ $\left(-1\leq D_{C}\leq 1\right)$; and the net depolarization Δ (0≤Δ≤1).

### 2.3 Samples

The experimental setup and the Mueller matrix calculation were verified by analyzing samples with well-defined Mueller matrices. Muller matrix images were measured in the absence of a sample (i.e., analyzing air), of an LP (Thorlabs, CCM1-PBS252/M), and of a quarter waveplate (Thorlabs, WPQ10M-850). The polarimeter’s measurements of optical anisotropy (linear retardance and its associated fast axis orientation) were verified with a Berek variable waveplate (Newport, 5540). This device enabled arbitrary setting of these properties within the polarimeter measurement range.

To validate the polarimeter and logarithm decomposition, an optical phantom exhibiting retardance and depolarization properties simultaneously (as opposed to ordered sequentially) was required. Polymer-based phantoms that exhibit strain-induced birefringence have been used to validate polarimeters and their associated methods.^32^^–^^36^ Validation was performed using tissue-mimicking optical phantoms constructed from polyacrylamide gels, as a base medium to allow strain-induced linear retardance, and an optically scattering media of lipid-based emulsion (Fresenius Kabi, Intralipid), to mimic tissue scattering properties. For validation, transparent and scattering phantoms were strained uniaxially and imaged to yield an estimate of linear retardance as a function of strain. The retardance-strain relationship for the transparent phantoms provided a reference for comparison with scattering phantoms to validate that optical anisotropy was correctly estimated in the presence of confounding depolarization.

Optical phantoms were constructed from polyacrylamide gels made with a 30% (w/v) acrylamide and N,N′-methylenebisacrylamide solution (ratio 37.5:1) polymerized using initiators of 0.1% (w/v) ammonium persulfate and 0.05% (v/v) N,N,N′,N′-tetramethylethylenediamine. The gels were polymerized in molds with cross-sectional dimensions of 10  mm×10  mm, cut to lengths of 35 to 45 mm, and stored at 4°C until used. Quasi-static uniaxial mechanical tests were performed on optical phantoms, and Mueller matrices were measured following each displacement step using a previously described mechanical testing instrument.^37^^–^^39^ Displacements were applied in steps of 0.1 mm to the gel along its length, at a velocity of 0.5  mm/s. A stress relaxation period of 15 s was provided prior to each Mueller matrix measurement.

Mueller matrix imaging was performed on a sample of calf bovine pericardium (Angus, 6 months) that had been stored at −86°C post-mortem and defrosted prior to imaging. 16 adjacent regions of the pericardium, of dimensions 14  mm×14  mm, were imaged. A preliminary optomechanical test was also performed on a sample of calf bovine pericardium to demonstrate optical polarization changes in these tissues, and assess practical limitations of the polarimeter. A 40  mm×10  mm sample was laser cut from a calf bovine pericardium, mounted, and then stretched uniaxially in displacement steps of 0.5 mm along its long-axis dimension, at a velocity of 0.5  mm/s. Following each displacement step, a pause of 45 s was made to account stress relaxation in the tissue, then a Mueller matrix measurement performed.

## 3 Results and Discussion

### 3.1 Verification

**Fig. 2:**
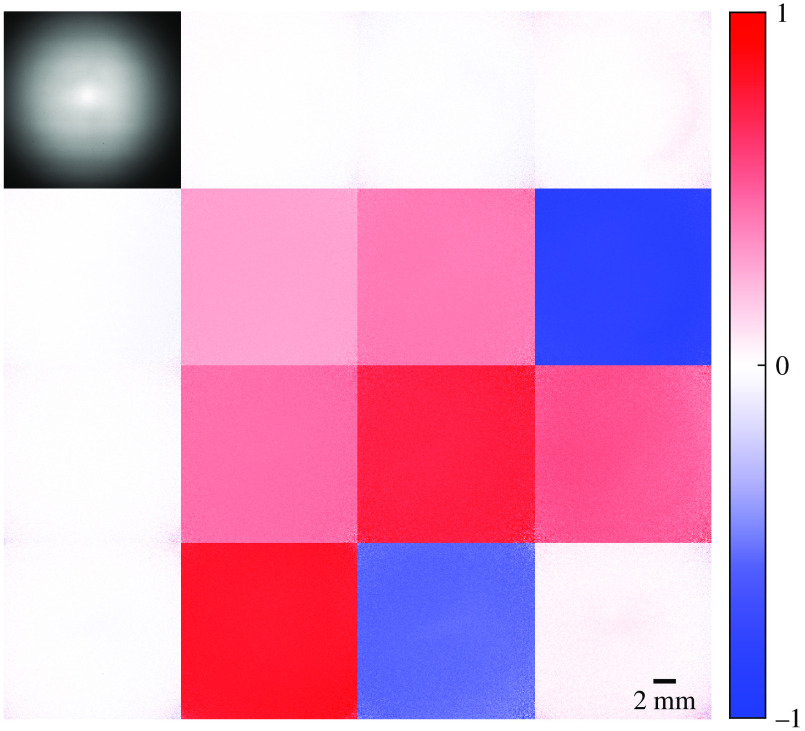
Mueller matrix images for a quarter waveplate with fast axis at 30 deg. The $m_{11}$ element shows the measured transmission of unpolarized light.

The theoretical and the measured Mueller matrices, averaged across the image, for samples with well-defined Mueller matrices are given in Table 1. The Mueller matrix images for the quarter waveplate are shown in Fig. 2. The measurements showed close agreement with associated theoretical values. The maximum absolute error for any Mueller matrix element was 0.063 and the root-mean-square error across all elements was 0.030 or less, for all samples.

**Table 1:** Verification of Mueller matrix measurement for polarimeter.

Theoretical Mueller matrix	Measured Mueller matrix (averaged over image)	Error	
		RMS	Max
Air			
$\left[\begin{array}{cccc} 1 & 0 & 0 & 0\\ 0 & 1 & 0 & 0\\ 0 & 0 & 1 & 0\\ 0 & 0 & 0 & 1 \end{array}\right]$	$\left[\begin{array}{cccc} 1.000 & -0.013 & -0.005 & -0.008\\ -0.013 & 0.999 & -0.005 & 0.036\\ -0.002 & 0.015 & 0.991 & 0.045\\ -0.021 & -0.001 & -0.031 & 0.940 \end{array}\right]$	0.024	0.060
Linear polarizer (0 deg)			
$\left[\begin{array}{cccc} 1 & 1 & 0 & 0\\ 1 & 1 & 0 & 0\\ 0 & 0 & 0 & 0\\ 0 & 0 & 0 & 0 \end{array}\right]$	$\left[\begin{array}{cccc} 1.000 & 0.996 & -0.018 & 0.039\\ 0.995 & 0.996 & -0.018 & 0.038\\ 0.016 & 0.016 & 0.002 & 0.000\\ -0.019 & -0.018 & -0.011 & -0.008 \end{array}\right]$	0.018	0.039
Quarter waveplate (30 deg)			
$\left[\begin{array}{cccc} 1 & 0 & 0 & 0\\ 0 & 0.250 & 0.433 & -0.866\\ 0 & 0.433 & 0.750 & 0.500\\ 0 & 0.866 & -0.500 & 0 \end{array}\right]$	$\left[\begin{array}{cccc} 1.000 & -0.001 & -0.013 & 0.011\\ -0.014 & 0.293 & 0.411 & -0.817\\ -0.004 & 0.454 & 0.741 & 0.524\\ -0.006 & 0.803 & -0.550 & 0.034 \end{array}\right]$	0.030	0.063

**Fig. 3:**
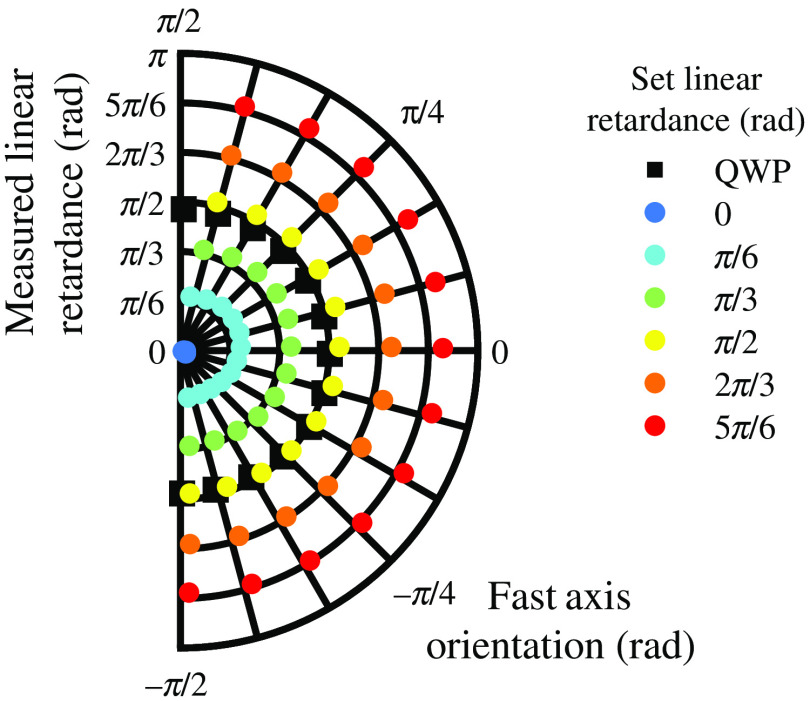
Verification of polarimeter linear retardance measurements. The measured linear retardance and fast axis orientation are shown for various settings of the variable waveplate and for the quarter waveplate.

The mean linear retardance and circular mean fast axis orientation, averaged across the variable waveplate’s clear aperture, are shown in Fig. 3. The measurements of the quarter waveplate (from Table 1) at the same fast axis orientations are also provided.

The quarter waveplate measurements showed close agreement with expected values. The maximum and mean absolute error in the fast axis orientation were 0.028 rad and 0.017 rad, respectively. This error was mainly attributed to the manual positioning of the quarter waveplate fast axis. The maximum and mean absolute error in the linear retardance were 0.075 and 0.040 rad, respectively. This error was attributed to non-ideal polarization elements in the polarimeter.

The measurements of linear retardance and fast axis orientation for the variable waveplate showed close agreement with the prescribed values. The measured fast axis orientation showed a linear relationship with the prescribed orientation with correlation ≥0.9994 for all linear retardance settings (excluding zero retardance). It is noted that there was a systematic deviation from the prescribed orientation of 0.070 rad, this was attributed to an incorrect calibration of the Berek variable waveplate fast axis. The maximum and mean absolute error in the linear retardance across all settings were 0.171 rad and 0.055 rad, respectively. A possible issue and source of error with the variable waveplate is that it controls retardance by tilting an optic axis out-of-plane, therefore violating the in-plane sample optic axis assumption of the forward scattering polarimeter in this study. Considering that the light source in the polarimeter had a Gaussian spatial intensity profile, this could give spatially mis-registered intensities across the camera sensor due to double refraction, leading to misinterpretations in linear retardance measurements. This explanation was reinforced by observing that errors were of the largest magnitude in the center of the polarimeter’s field of view. A variable waveplate with in-plane optic axis, such as a Soleil–Babinet compensator or a liquid crystal variable retarder, may offer a better alternative for future verifications.

### 3.2 Optical Phantoms

**Fig. 4:**
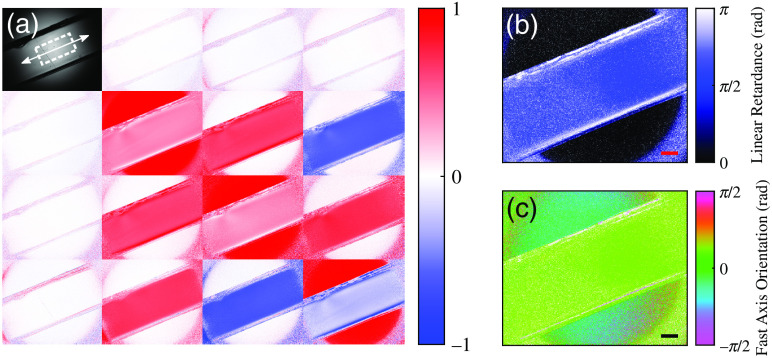
Mueller matrix images (a) and decomposed properties of linear retardance (b) and fast axis orientation (c) for a transparent polyacrylamide optical phantom at 6% strain. The direction of positive strain is indicated by white arrow in the $m_{11}$ element of (a). Scale bars represent 2 mm. Undefined regions are displayed as black for (b) and white for (c).

Two transparent optical phantoms were mechanically strained and imaged. The Mueller matrix images and the corresponding decomposed linear retardance and fast axis orientation for a transparent phantom, at 6% strain, are shown in Fig. 4. Linear retardance was homogenous across the phantom and the measured fast axis was aligned along the direction of strain with circular mean $\theta_{\mathrm{FA}}=0.384\;\mathrm{rad}\pm 0.013\;\mathrm{rad}$ for the step shown in Fig. 4(c) (the phantom was mounted and strained on an axis at 0.393 rad in the mechanical tester).

**Fig. 5:**
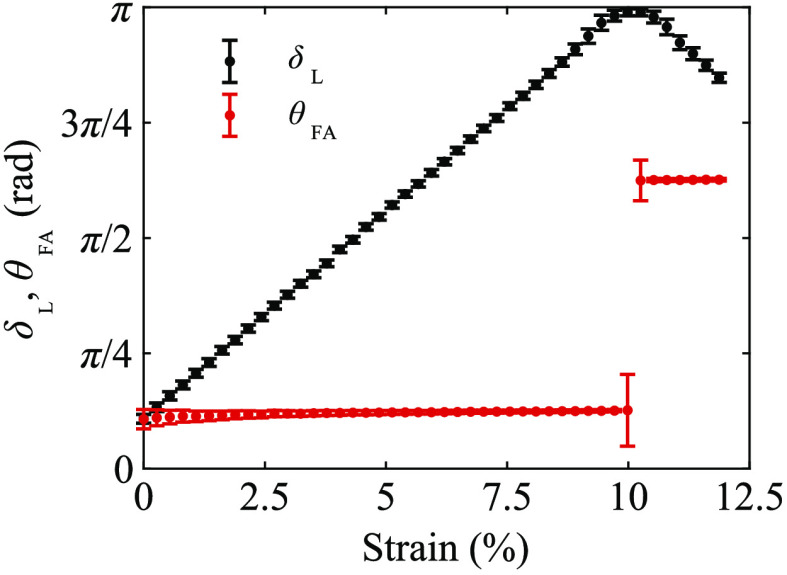
Measured linear retardance and fast axis orientation (averaged over the region of interest indicated in Fig. 4(a) as a function of strain for a transparent polyacrylamide optical phantom. Error bars indicate ±1 SD for the region of interest.

The relationship between measured linear retardance $\left(\delta_{L}\right)$, averaged over a central region [indicated by the white dashed rectangle in the $m_{11}$ element of Fig. 4(a)], and the strain (ε) of the transparent phantom is shown in Fig. 5. Considering only strains with $\delta_{L}<\pi$, the two transparent phantoms exhibited a linear relationship between measured linear retardance and strain with $\delta_{L}=28.2\varepsilon +0.3$ $\left(R^{2}=0.9999\right)$ and $\delta_{L}=27.6\varepsilon +0.1$ $\left(R^{2}=0.9999\right)$. At higher strains, the measured linear retardance showed phase wrapping (due to a π periodicity) to (π,0] for sample retardance $\pi \leq \delta_{L}<2\pi$. This wrapping was also associated with an offset in the fast axis orientation of π/2 (i.e., perpendicular to the strain direction). Measurements with the polarimeter were therefore limited to a total retardance (linear and circular) of π radians for unambiguous optical anisotropy estimates. The wrapping effects could be corrected with knowledge of the sample’s retardance half-wave order ($0\leq \delta_{L}<\pi$ or $\pi \leq \delta_{L}<2\pi$). For the optical phantoms used in this study, the half-wave order could be safely assumed, from the linear retardance to strain relationship, to be second-half order beyond strains of ∼10%. It should be noted that some linear retardance was exhibited in the phantoms at zero recorded strain as they were not mounted in a completely unstrained state.

**Fig. 6:**
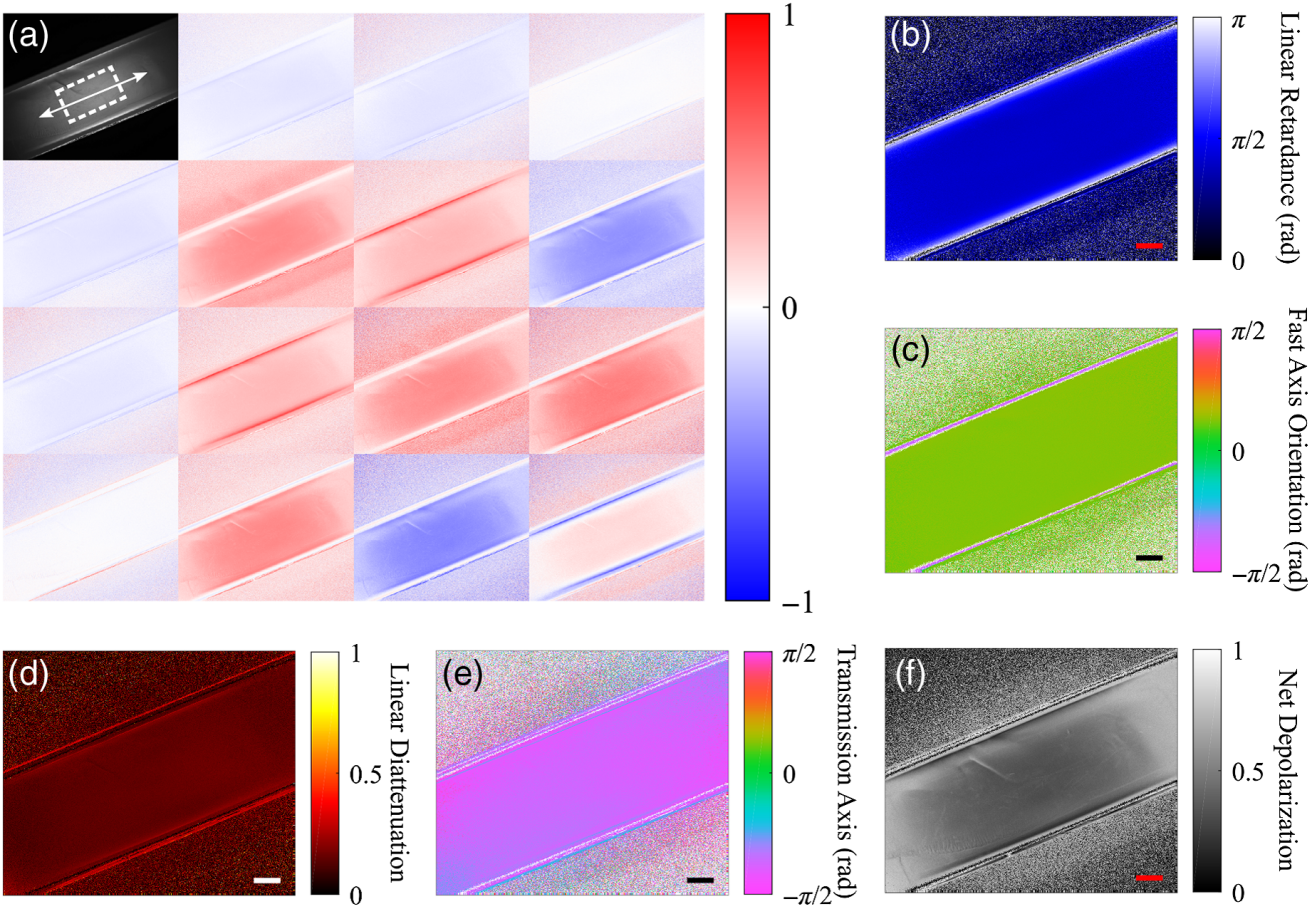
Mueller matrix images (a) and decomposed properties of linear retardance (b), fast axis orientation (c), linear diattenuation (d), transmission axis orientation (e), and net depolarization (d) for a scattering phantom with 1% lipid at 6% strain. The direction of positive strain is indicated by the white arrow in the $m_{11}$ element of (a). Scale bars represent 2 mm. Undefined regions are displayed as black for (b), (d), and (f), and white for (c) and (e).

Two scattering optical phantoms with lipid concentrations of 0.75% and 1% were mechanically strained and imaged. The Mueller matrix images and the observed polarization properties for the scattering phantom with 1% lipid, at 6% strain, are shown in Fig. 6. The phantom with 1% lipid concentration yielded physiologically relevant values for net depolarization of ∼0.4 at its initial unstrained state, this was similar to that seen in pericardial membrane tissue presented in Sec. 3.3. The scattering phantoms exhibited both a strain-dependent linear retardance, with the fast axis orientation parallel to the direction of strain, and a strain-dependent linear diattenuation, with the transmission axis orientation orthogonal to the direction of strain.

**Fig. 7:**
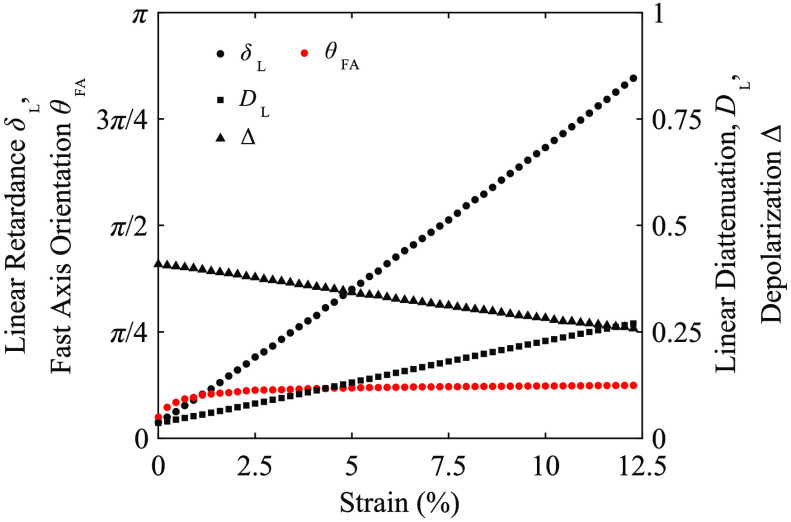
Measured polarization properties (averaged over the region of interest indicated in Fig. 6(a) as a function of strain for a scattering optical phantom with 1% lipid. Error bars have been omitted for clarity.

The relationship between strain (ε) and the measured linear retardance $\left(\delta_{L}\right)$, fast axis orientation, linear diattenuation, and net depolarization averaged over a central region [indicated in Fig. 6(a)], is shown for the 1% lipid phantom in Fig. 7. The fast axis orientations for both scattering phantoms showed close agreement with the strain direction. This indicated the polarimeter correctly estimated optical anisotropy in samples with simultaneous depolarization similar to that observed in tissues. Fitting a linear relationship to the measured linear retardance gave $\delta_{L}=21.4\varepsilon +0.03$ $\left(R^{2}=0.9992\right)$ and $\delta_{L}=20.8\varepsilon +0.08$ $\left(R^{2}=0.9995\right)$ for the 0.75% and 1% lipid concentration phantoms, respectively, indicating a strong linear relationship, as was observed for the transparent phantoms.

### 3.3 Pericardium

**Fig. 8:**
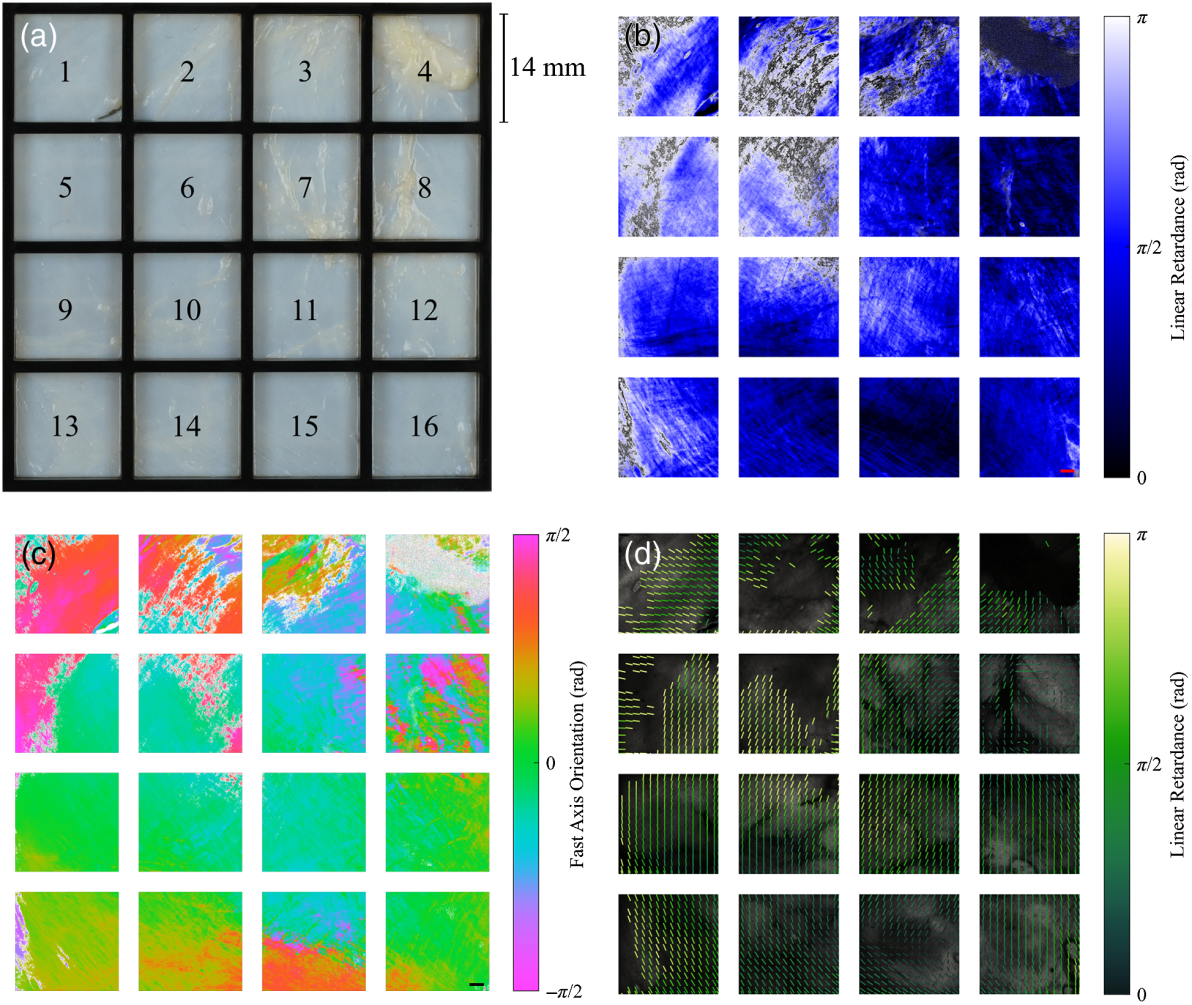
(a) Calf bovine pericardium with imaged regions labeled 1 to 16. (b) Linear retardance; (c) fast axis orientation; and (d) optical anisotropy maps for regions 1 to 16 of the sample. The scale bars represent 2 mm. Undefined regions are displayed as black for (b) and white for (c). For the optical anisotropy map, lines in each subregion (∼1  mm×1  mm) are oriented with the local circular mean of the (slow) optic axis orientation, and length is normalized by the corresponding local circular variance. Subregions with more than 25% undefined values have been omitted.

Mueller matrix imaging of the 16 adjacent regions of the pericardium is shown in Fig. 8 together with the corresponding observed polarization properties. The maps of linear retardance and fast axis orientation for each tissue region are shown in Figs. 8(b) and 8(c), respectively. The linear retardance and fast/slow axis orientation can be combined into more intuitive maps of optical anisotropy for these tissues by considering that type I collagen has positive uniaxial birefringence and, therefore, has a slow optic axis parallel to its fiber axis. Maps of tissue optical anisotropy are displayed in Fig. 8(d) as overlaid lines in each subregion (∼1  mm×1  mm), with orientation and length corresponding to the local circular mean of the (slow) optic axis orientation and normalized local circular variance, respectively.

Optical anisotropy results indicated that the membrane had average fiber orientations that were generally homogeneous within the imaged 14  mm×14  mm regions, but with varying predominant orientations across the larger 70  mm×70  mm section of the membrane that was analyzed. This result indicates that uniform regions can be identified in these tissues using Mueller matrix imaging. Since this imaging technique is non-destructive, this also demonstrates the potential for its application in tissue selection, for example, for the manufacture of bioprosthetic heart valves. It has been suggested that regions of heterograft membranes with structurally uniform fiber orientations should be selected and oriented such that the fiber direction aligns with the circumferential direction of the leaflets to mimic the mechanical response of native heart valves.^16^ It is noted that there are potential limitations and ambiguities in the optical anisotropy measurements in this study, which are discussed below.

The differential matrix formalism used to calculate the Mueller matrix logarithm and the associated polarization properties assumed transverse and longitudinal homogeneity in the medium. Practically, this assumes that the pericardial membrane is optically homogeneous in the volumes of tissue corresponding to each pixel. The validity of the homogeneity assumption will therefore likely depend on the spatial resolution of the Mueller matrix, which corresponds to the optical resolution of the polarimeter, and any tissue microstructural variations through the membrane thickness. Mueller matrix measurements of optical anisotropy are yet to be directly correlated to structural anisotropy in these samples. It is evident that this correlation is a necessary step to link the non-destructive imaging technique in this study to soft tissue structure and mechanical function relationships. To the authors’ knowledge, no full-volume studies have been previously performed for bovine pericardium membranes. Recent work combining Mueller matrix imaging with second harmonic generation and two-photon excitation fluorescence as ground truths for structures of collagen and elastin, respectively,^40^^,^^41^ may offer a promising solution for correlating measurements from the Mueller matrices to fibrous components in soft tissue membranes.

The retardance of light propagating through the sample may be induced by both scattering and birefringence.^42^ As the polarimeter in this study is not capable of distinguishing the cause of the phase shift, the relative contribution of these two mechanisms is unknown. A test was performed by optically clearing a sample of pericardial tissue (Ce3D, 24 h). The resulting Mueller matrix measurements (results not shown) indicated that the mean linear retardance across the sample was reduced by ∼50%. Although the sample exhibited shrinking due to the clearing process, the predominant fast axis orientation of linear retardance over the sample was very similar to that of the uncleared tissue. This suggests that the optic axis orientation provides a reliable measure of the axis of optical anisotropy (due to birefringence) in these tissues, although a larger study is recommended to confirm this.

The pericardial sample exhibited high spatial variability in the measured linear retardance across the imaged regions, ranging from zero retardance up to the retardance limit of π. In some regions, particularly 1, 2, 3, and 5, it was observed that the tissue exhibited a high linear retardance associated with an abrupt π/2 change in the measured fast axis orientation with neighboring tissue, suggesting that the pericardium in these regions exceeded the polarimeter’s retardance measurement limit. The phase wrapping that occurred beyond this limit makes interpretation of optical anisotropy properties potentially ambiguous for these tissue areas. The polarimeter in this study was designed for use with near-infrared wavelengths, as opposed to visible wavelengths, to achieve both deeper penetration and larger measurable optical path differences in tissue samples. The latter gives linear retardances of lower magnitude compared to shorter wavelengths and thus achieves a higher retardance measurement limit. This design was shown to be sufficient for the majority of the imaged regions for the sample of calf bovine pericardium. However, some areas were beyond the retardance measurement limit of the polarimeter, leading to phase wrapping and thus ambiguity in the fiber orientation estimates. While imaging polarimetry allows manual correction of ambiguous regions, by observing spatially abrupt changes in the optic axis that are physiologically questionable, a more rigorous method to identify or possibly even avoid these ambiguities is recommended. Possible approaches for resolving these ambiguities include: an additional Mueller matrix measurement at a different wavelength; and/or at a different sample depth position (or thickness).

**Fig. 9:**
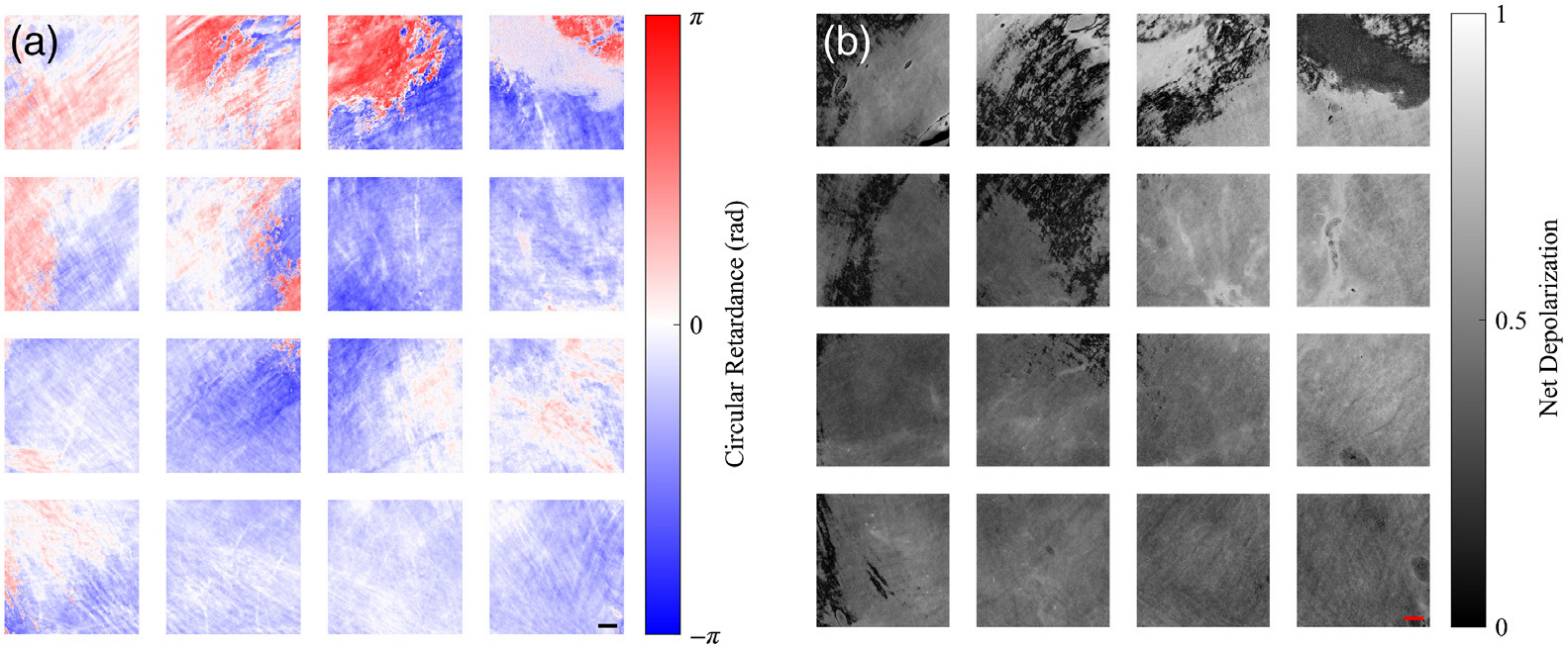
(a) Circular retardance and (b) net depolarization maps for regions 1 to 16 of the calf bovine pericardium. The scale bars represent 2 mm. Undefined regions are displayed as white for (a) and black for (b).

Circular retardance varied across the membrane with observed values that were generally negative, with values ranging from ∼0 to −1  rad [see Fig. 9(a)]. The large positive circular retardance occurring in regions 1 to 6 was likely associated with the wrapping effects due to total retardance of the tissue exceeding the polarimeter’s measurement limit. These locations of abrupt sign change are consistent with the corresponding effects seen for linear retardance and its associated fast axis orientation. While the effect of circular retardance in biological samples has generally been reported to be weak,^31^ we observed strong circular retardance in calf bovine pericardium. The source of this circular retardance in these tissues is currently unknown. Mueller matrix measurements conducted before and after optically clearing a sample of pericardial tissue showed a complete loss of the measured circular retardance. This suggests that the mechanism for circular retardance in these tissues is due to scattering, as opposed to circular birefringence.

The net depolarization maps for each tissue region are shown in Fig. 9(b). Mueller matrix imaging of calf bovine pericardium indicated that significant depolarization of light occurred across the membrane, with regional means ranging from 0.31 to 0.73. Generally, this tissue did not cause complete depolarization of light, although particularly significant depolarization occurred in the upper right tissue regions and complete depolarization occurred in locations for which adherent loose connective tissue was present (regions 4, 7, and 8). These measurements indicated that the Mueller matrix imaging polarimeter developed for this study was sufficient to non-destructively characterize these soft tissue membranes without complete loss of polarization information. Mueller matrix imaging of calf bovine pericardium showed that this tissue exhibits low diattenuation properties, with logarithm decomposition giving mean linear diattenuation values of between 0.08 and 0.16 for all regions.

**Fig. 10:**
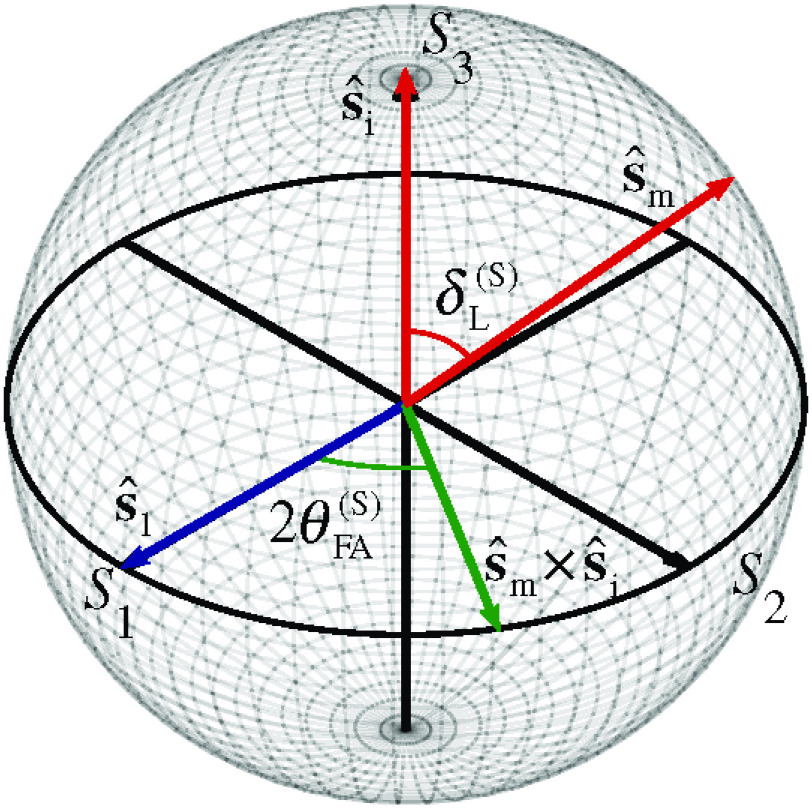
Poincaré sphere transformation for a Stokes sample measuring polarimeter with calculation of linear retardance and fast axis orientation.

The confounding effect of the other polarization properties on optical anisotropy measurements was considered by comparing the Mueller polarimeter measurements to a naïve approach that assumes tissue samples with linear retardance properties only. A previous approach using transformations of normalized Stokes vectors in the Poincaré sphere from a complete Stokes sample measuring polarimeter was adopted and is described here.^43^ The fast axis of a pure linearly retarding sample lies in the $S_{1}-S_{2}$ plane in the Poincaré sphere. The linear retardance and fast axis orientation can be calculated from the transformation of the Stokes vector for incident light s^i to the measured Stokes vector for output light s^m (see Fig. 10). The linear retardance $\delta_{L}^{\left(S\right)}$ can be modeled as the rotation angle about the fast axis that transforms s^i onto s^m,^44^^,^^45^ calculated as^43^^,^^46^^,^^47^

δL(S)=cos−1(s^i·s^m|s^i||s^m|)0≤δL(S)<π. (2)

The sample fast axis in the Poincaré sphere is given by the vector normal to the plane containing s^i and s^m, and thus the fast axis orientation $\theta_{\mathrm{FA}}^{\left(S\right)}$ can be calculated by the angle between their cross product and the unit vector s^1, aligned with the reference axis ($S_{1}$ in the Poincaré sphere), as

θFA(S)=±12 cos−1((s^m×s^i)·s^1|s^m×s^i||s^1|)−π2<θFA(S)≤π2, (3)

where the sign of $\theta_{\mathrm{FA}}^{\left(S\right)}$ is the same sign as the $S_{2}$ component of s^m×s^i.^46^

By considering the illumination of samples with circularly polarized light only, the calculated linear retardance is independent of the sample fast axis orientation.^44^^,^^48^ As such, right circularly polarized incident light with s^i=[0,0,1]T was used. The measured Stokes vector for incident right circularly polarized light s^m can be calculated from the measurements (described in Eq. 1) as

s^m=1I0[2IRH−I02IRP−I02IRR−I0], (4)

where $I_{0}=I_{\mathrm{RH}}+I_{\mathrm{RV}}$ is the total intensity of emitted light for input right circularly polarized light.

**Fig. 11:**
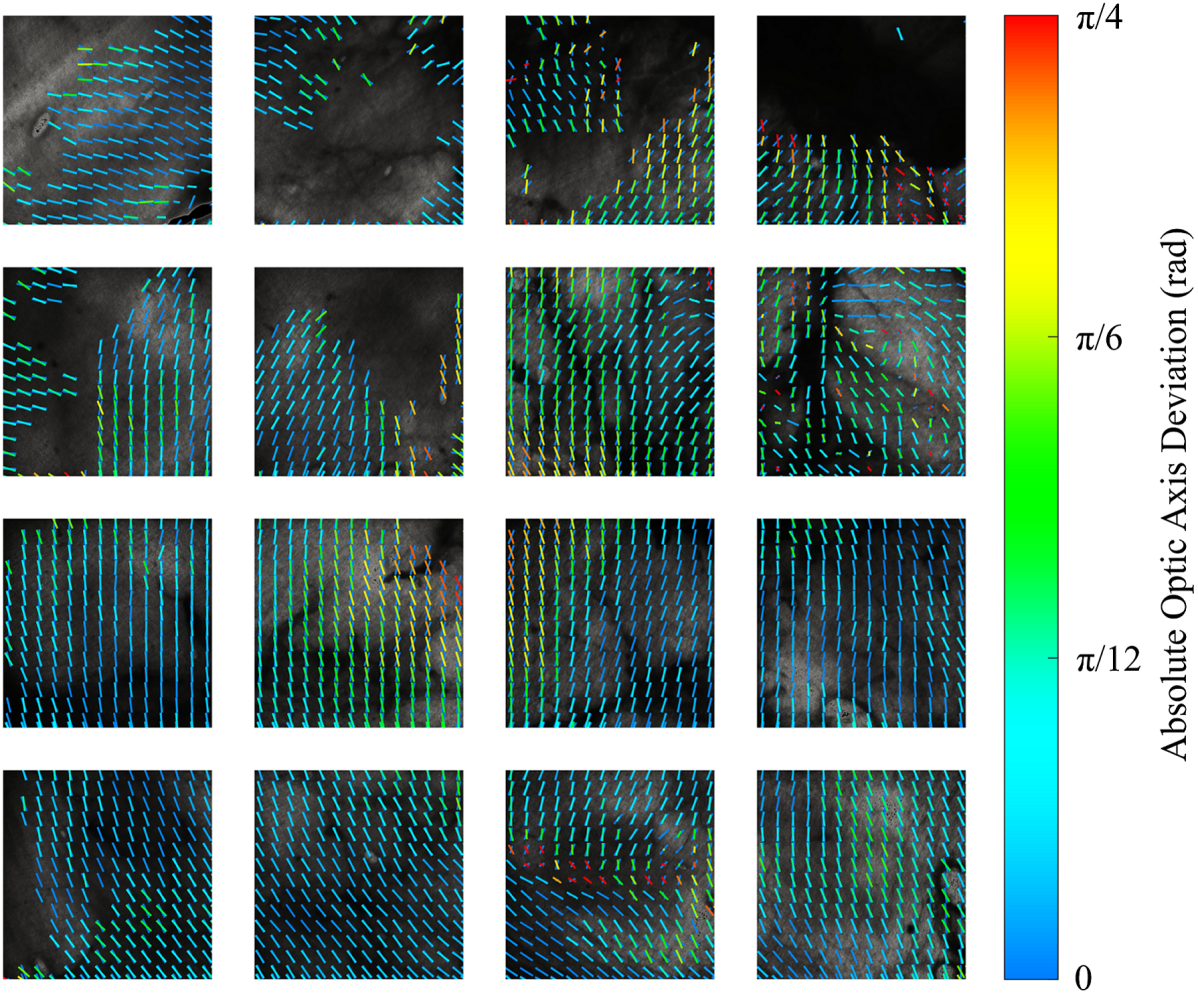
Estimation of optic axis deviation with Stokes polarimeter measurements compared to Mueller polarimeter estimates for regions 1 to 16 of the calf bovine pericardium. Line segments in each subregion (1  mm×1  mm) are oriented with the local circular mean of the (slow) optic axis orientation and length normalized by the corresponding local circular variance. Color indicates the absolute deviation of the Stokes polarimeter optic axis from the Mueller polarimeter optic axis (shown for reference with color of zero deviation). Note: the color range was limited to deviations in the range [0,π/4]  rad for clarity.

Deviations in measurements of the optic axis orientation, due to misinterpretation by the Stokes polarimeter, were estimated by comparing the linear retardance, $\delta_{L}^{\left(S\right)}$, and fast axis orientation, $\theta_{\mathrm{FA}}^{\left(S\right)}$, calculated according to Eqs. (2) and (3), respectively, with the $\delta_{L}$ and $\theta_{\mathrm{FA}}$ calculations based on logarithmic decomposition of the Mueller matrix. The estimated absolute optic axis deviation is shown in Fig. 11 with overlaid maps of anisotropy for both methods. There were large deviations in the measured optic axis orientation using the Stokes polarimeter. These deviations varied over the sample and were particularly apparent in the pericardial regions that exhibited greatest circular retardance [see Fig. 9(a)].

**Fig. 12:**
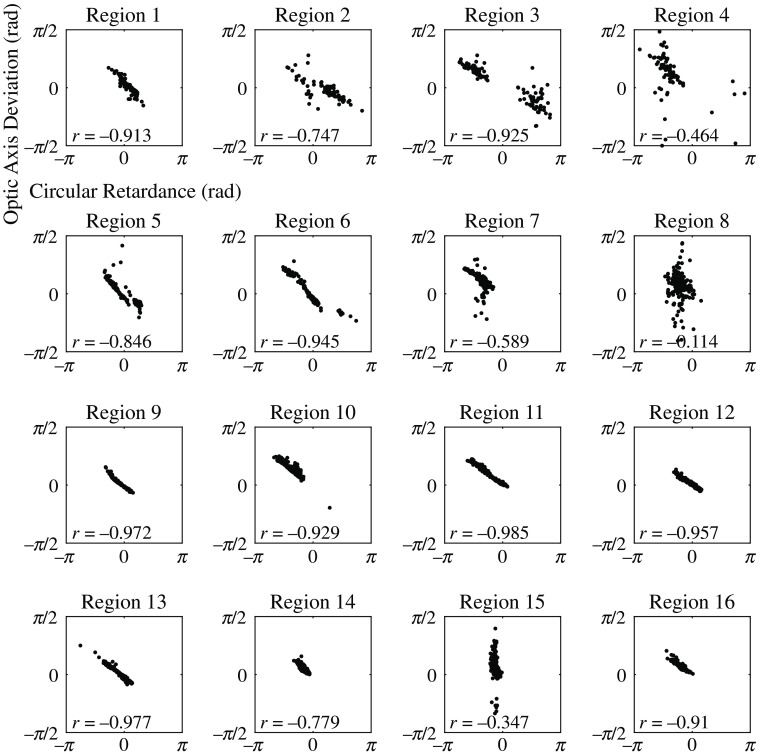
Stokes polarimeter deviation in the optic axis orientation as a function of circular retardance, averaged over 1  mm×1  mm subregions, for regions 1 to 16 of the calf bovine pericardial sample. The Pearson correlation coefficient (r) is given for each region.

The relationship between optic axis deviation and circular retardance is shown for each tissue region in Fig. 12. A strong negative linear correlation was observed for most tissue regions. The deviation of the optic axis can be explained by considering that a circularly retarding medium will rotate the incident Stokes vector s^i about the $S_{3}$ axis in the Poincaré sphere (see Fig. 10), thus giving an apparent optic axis orientation that deviates from that expected due to linear retardance. The deviation is negatively correlated as the rotation about $S_{3}$ is clockwise for positive circular retardance, and the deviation angle is half the circular retardance, as angles in the Poincaré sphere are twice that in physical space. No strong dependence was observed for optic axis deviation with respect to linear diattenuation or depolarization properties.

As this tissue exhibited significant circular retardance, which was transversely heterogeneous across the membrane, it is concluded that measurement of circular retardance is necessary to avoid large misinterpretations in optical anisotropy measurements of these tissues. It is noted that in previous studies using a widely used polarization imaging technique, commonly referred to as quantitative polarized light microscopy (QPLM), the collagen fiber architecture has been estimated in soft tissue membranes of native heart valve assuming these samples exhibit linear birefringence only.^49^^,^^50^

**Fig. 13:**
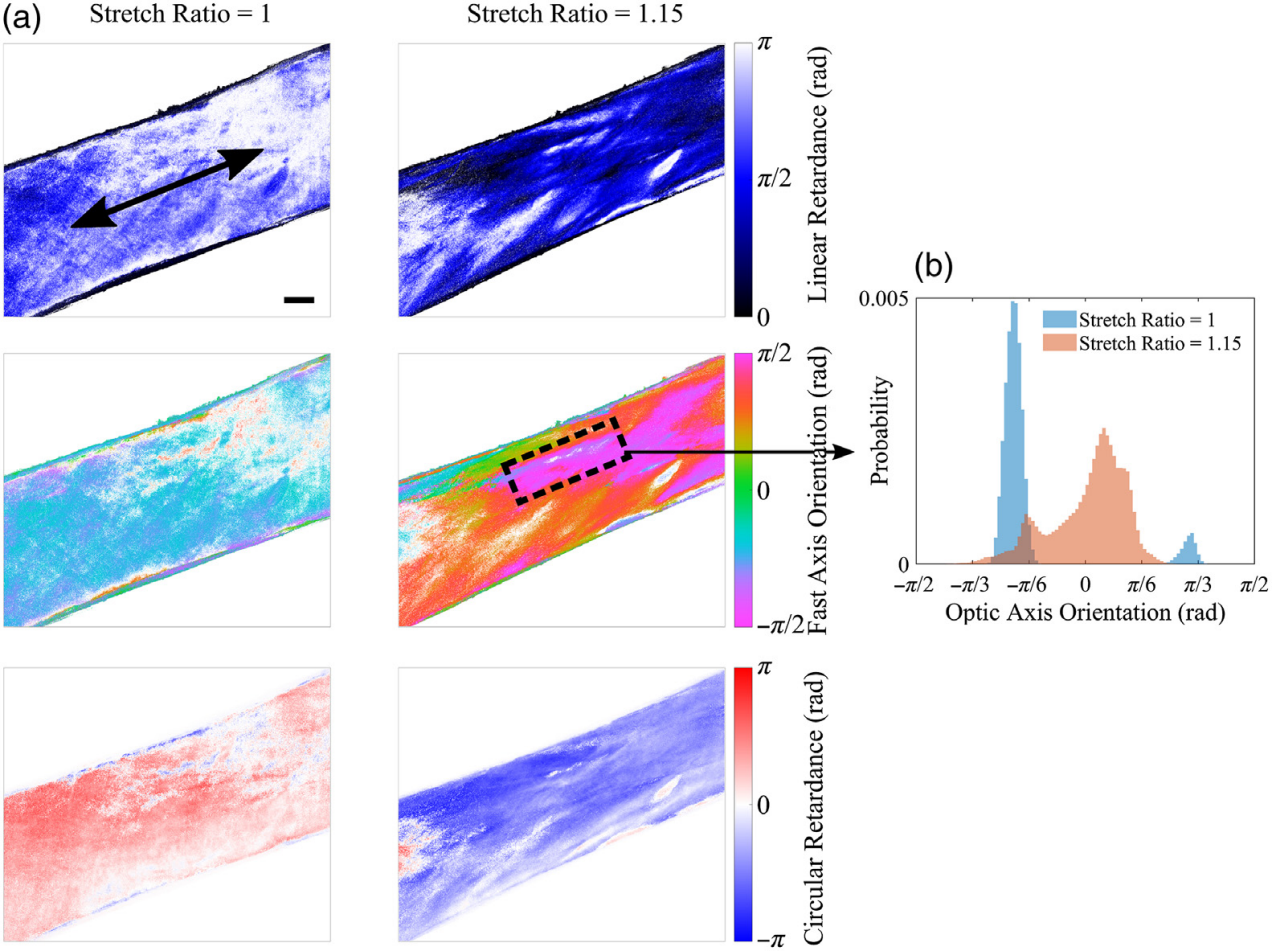
(a) Retardance polarization properties of linear retardance, fast axis orientation, and circular retardance for the sample of calf bovine pericardium mounted in a uniaxial tester at stretch ratios of 1 and 1.15. The sample is mounted at +22.5  deg to the horizontal axis. Undefined regions (from the logarithm decomposition) are displayed as white for all properties. In the top-left map, the direction of stretch is indicated by the black arrow and the black scale bar represents 2 mm. (b) Histograms of the distribution of assumed optic axis orientations at both stretch states within the region of the tissue indicated by the dashed rectangle.

The retardance polarization properties of the pericardial sample from the preliminary optomechanical test at two states of stretch - a stretch ratio of 1 (unloaded) and a stretch ratio of 1.15—are shown in Fig. 13(a). The pericardium exhibited large changes in linear retardance when stretched. These changes were of higher magnitude than those observed for circular retardance (ignoring the sign change due to phase wrapping). This mechanism is likely due to strain-induced changes in the linear birefringence of the tissue associated with reorganization of the collagen fiber architecture in response to loading. In addition to intrinsic linear birefringence, tissues containing type I collagen can exhibit a positive uniaxial form linear birefringence that arises due to the parallel arrangement of fibrils embedded in the ground substance of the extracellular matrix, which exhibits a lower refractive index than collagen.^5^^,^^18^ As networks of collagen become aligned or dispersed in different regions during loading of the tissue, the form birefringence will increase or decrease, respectively,^42^ leading to large changes in measured linear retardance.

Phase wrapping effects were apparent, in particular, during the mechanical stretch of the pericardium sample, where abrupt changes in the fast axis orientation and circular retardance, due to the fluctuations in linear retardance, were observed between the unloaded and stretched states of the tissue. This made interpretation of the optical anisotropy in the sample ambiguous, as the true retardation at each stretch state is unknown. A physical interpretation of the retardance measurements can be made by considering that there are two possible cases for the sample’s total retardance, $\delta_{T}=\sqrt{\delta_{L}^{2}+\delta_{C}^{2}}$: it is within the first half of a retardance order, that is $2p\pi \leq \delta_{T}<(2p+1)\pi$, where p=0,1,2,… or within the second half of a retardance order, that is $(2p+1)\pi \leq \delta_{T}<2(p+1)\pi$. The latter case gives a measured fast axis that is incorrectly offset by π/2. The optic axis orientation can be corrected for the unloaded state by assuming that the unloaded sample’s total retardance lies in the second half of a retardance order, and that, when stretched, most regions of the tissue shift to the first half of a retardance order, due to strain-induced changes in birefringence. Under these assumptions, these results indicate that the tissue exhibited an optical anisotropy in the unloaded state that was homogeneous across the sample, with optic axis orientation predominantly at −40  deg. Upon stretching, the tissue exhibited optic axis orientations that were heterogeneous across the sample, and in some regions realigned toward the direction of stretch. This is shown in Fig. 13(b), with histograms of the distributions of optic axis orientations for the two states of stretch, within a region of the tissue (indicated by the dashed rectangle). This indicated that the linearly birefringent fibrous components of the tissue, such as collagen, which is the main load bearing structure in soft tissues, underwent realignment toward the direction of stretch. The previous assumptions of retardance order are therefore consistent with the expected biomechanical behavior of the tissue.

These preliminary optomechanical results demonstrate the potential of this technique for understanding the mechanical behavior of industry standard bioprosthetic heart valve materials, such as calf bovine pericardium. The phase wrapping in retardance was particularly problematic during simultaneous Mueller matrix imaging and mechanical testing, where significant strain-induced changes in the tissue retardance were observed. This gave rise to ambiguous apparent fiber orientations without careful assumptions of the retardance order. An objective approach for resolving these ambiguities is required for meaningful interpretation of measurements in future structure-function studies with these tissues. We plan to address this in future studies with Mueller matrix measurements using more than one illumination wavelength.

## 4 Conclusion

In this study, we presented a new macroscopic Mueller matrix imaging polarimeter for transmission imaging of soft tissue membranes. The complete polarization properties of samples were estimated from Mueller matrix measurements using a logarithm decomposition that assumed simultaneous and continuous occurrence of these properties across the thickness of the tissue as opposed to a particular order of retarder, diattenuator, and depolarizer components. Measurements from the polarimeter were validated using samples with well-defined optical properties. Mueller matrix imaging of calf bovine pericardium indicated that significant (but not complete) depolarization of light occurred across the membrane. Mueller matrix imaging showed, to the authors’ knowledge, the first reported measurement of significant circular retardance in these tissues. It was demonstrated that this circular retardance would lead to large deviations in the estimated fiber orientation if left unaccounted for using conventional widely used polarization analyses, such as QPLM. This has significant implications for transmission polarization imaging of tissue membranes, such as pericardium and heart valves, where previous studies have estimated collagen fiber architecture using techniques that assume the tissues exhibit only linear birefringence. The use of Mueller matrix imaging and analysis addresses these shortcomings. In the present study, optical anisotropy measurements were able to identify regions of pericardial tissue with fiber orientations that were generally uniform. This non-destructive imaging device may enable more reliable selection of suitable regions of soft tissue membranes for applications such as the manufacture of bioprosthetic heart valves.
